# Polymeric Drug Delivery System Based on Pluronics for Cancer Treatment

**DOI:** 10.3390/molecules26123610

**Published:** 2021-06-12

**Authors:** Jialin Yu, Huayu Qiu, Shouchun Yin, Hebin Wang, Yang Li

**Affiliations:** 1 College of Material, Chemistry and Chemical Engineering, Hangzhou Normal University, Hangzhou 311121, China; yujialin1@stu.hznu.edu.cn (J.Y.); hyqiu@hznu.edu.cn (H.Q.); yinsc@hznu.edu.cn (S.Y.); 2 Key Laboratory of Organosilicon Chemistry and Materials Technology of Ministry of Education, Hangzhou Normal University, Hangzhou 311121, China; 3 College of Chemical Engineering and Technology, Tianshui Normal University, Tianshui 741099, China

**Keywords:** pluronic, hydrophilic-lipophilic balance, cancer therapy, drug delivery system, stimulus-responsive polymer

## Abstract

Pluronic polymers (pluronics) are a unique class of synthetic triblock copolymers containing hydrophobic polypropylene oxide (PPO) and hydrophilic polyethylene oxide (PEO) arranged in the PEO-PPO-PEO manner. Due to their excellent biocompatibility and amphiphilic properties, pluronics are an ideal and promising biological material, which is widely used in drug delivery, disease diagnosis, and treatment, among other applications. Through self-assembly or in combination with other materials, pluronics can form nano carriers with different morphologies, representing a kind of multifunctional pharmaceutical excipients. In recent years, the utilization of pluronic-based multi-functional drug carriers in tumor treatment has become widespread, and various responsive drug carriers are designed according to the characteristics of the tumor microenvironment, resulting in major progress in tumor therapy. This review introduces the specific role of pluronic-based polymer drug delivery systems in tumor therapy, focusing on their physical and chemical properties as well as the design aspects of pluronic polymers. Finally, using newer literature reports, this review provides insights into the future potential and challenges posed by different pluronic-based polymer drug delivery systems in tumor therapy.

## 1. Introduction

**Figure 1:**
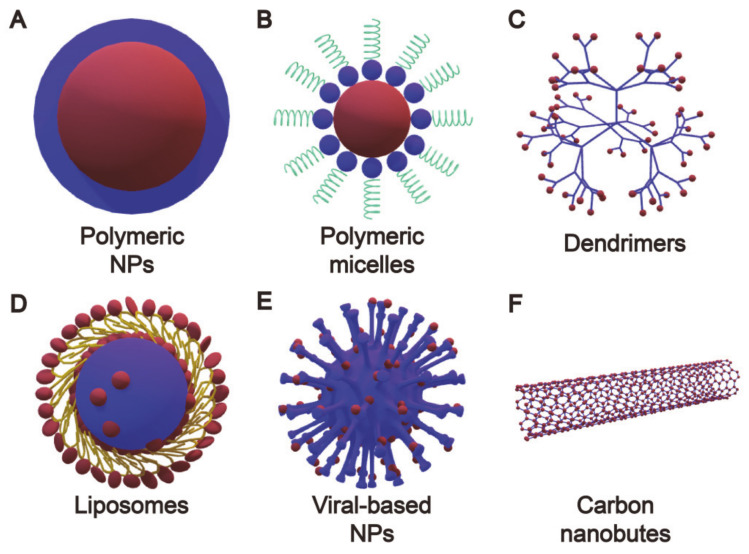
Types of nanocarriers for drug delivery.

Cancer remains one of the biggest “first killer” diseases in the world [1,2,3]. Currently, clinical cancer treatment involves surgery, chemotherapy, and radiotherapy [4,5,6]. As a local therapy, there are difficulties using surgical treatment to eliminate infiltrating and metastatic tumors, while chemotherapy often results in serious side effects, including organ damage, nausea, vomiting, leukopenia, and anemia, as well as limited drug accumulation in deep tumors that can lead to drug resistance after repeated treatment [7,8,9]. Recently, methods to minimize the side effects of chemotherapy on normal tissues have attracted widespread attention [10,11,12,13]. Multifunctional drug delivery systems (DDSs) act as drug carriers that are designed to deliver a sufficient dose of a drug to the tumor area at the required rate, and are widely used today [9,14,15]. DDSs can effectively prolong the circulation time of drugs in the body, while reducing side effects and increasing the accumulation of drugs at the tumor site through infiltration of the tumor interstitial tissue from leaky vasculature [16,17]. Drug carriers are essential to obtain good therapeutic effects, prolong blood circulation time, improve loading efficiency, and control release capabilities [18]. Therefore, the most important feature for drug carriers is excellent biocompatibility, which refers to the ability of tissues to react to inactive materials, and generally refers to the compatibility between the material and the host. Good biocompatibility is also conducive to a material’s applications in biomedicine. There are many types of drug carriers in a drug delivery system, including (1) polymeric nanoparticles, (2) polymeric micelles, (3) dendrimers, (4) lipid-based drug carriers, (5) viral nanoparticles, and (6) carbon nanotubes (Figure 1) [19].

Polyethylene glycol (PEG) is commonly used as the hydrophilic part of polymeric micelles because it is biocompatible and biodegradable, and has been approved for human use by the US Food and Drug Administration (FDA) [20,21,22]. A large fraction of nanoparticles may be sequestered and cleared by the reticulo-endothelial system (RES), but PEG may provide a stealth functionality for DDS because its addition can block serum protein binding to the surface of DDSs and inhibit their phagocytosis. Therefore, DDSs coated with PEG significantly delays macrophage-mediated clearance of nanoparticles and enhances their persistent circulation [23,24,25]. Although PEG prolongs the circulation time in the body, it can also affect the cellular uptake efficiency of a DDS [26,27]. The well-known triblock copolymers of oxyethylene (PEO) and oxypropylene (PPO) were commercialized as Pluronic^®^ polyols by Wyandotte Chemical Corp [28,29,30,31,32,33,34]. Pluronic^®^ polymers have been approved by the FDA for pharmaceutical use and have been investigated for different therapeutic applications due to their PEG hydrophilic chains and PPO hydrophobic units [35,36,37,38]. Due to their amphiphilic properties, these copolymers exhibit surfactant properties, including the ability to interact with hydrophobic surfaces and biofilms. When their concentration is greater than the critical micelle concentration (CMC), pluronic micelles can form spontaneously, which results in several beneficial properties: PPO blocks can be used to carry hydrophobic drugs, the hydrophilic PEO chain has a stabilizing effect, and the removal of the RES is avoided [39,40,41]. The hydrophilic part of the micelle provides steric stability and thus prolongs circulation time in the body, while the hydrophobic block copolymer has a high drug loading capacity and good compatibility [42]. Moreover, the hydrophobic block of the pluronic polymer can improve the efficiency of phagocytosis and promote drug absorption. Some pluronic polymers can also deplete intracellular adenosine triphosphate (ATP), inhibit the function of P-glycoprotein (P-gp), affect drug efflux and transport, and have the effect of reversing tumor multi-drug resistance (MDR) [43,44,45,46,47,48,49]. In this review, we focus on the latest research on various stimulus-responsive pluronic-based polymer drug delivery systems.

## 2. Pluronic Classification

With a wide range of applications in the pharmacutical field, pluronic polymers are often used as solubilizers, emulsifiers, matrices, solid dispersion carriers, absorption enhancers, and stabilizers drugs in order to increase their solubility, improve their stability, control their release, and improve their biological utilization [50,51].

**Figure 2:**
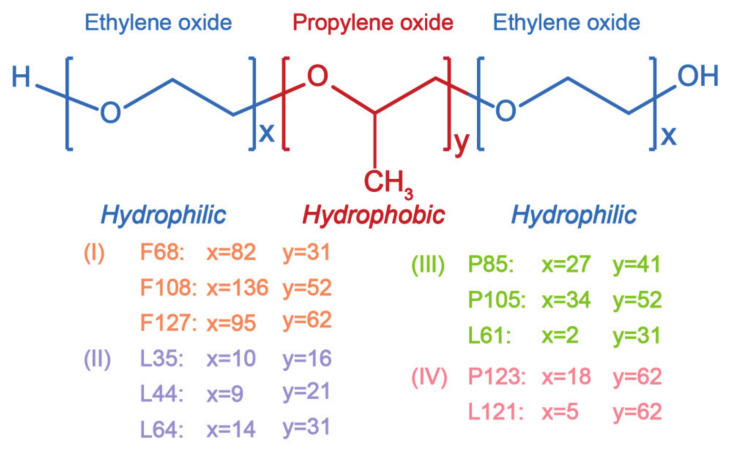
The structures of the different pluronic^®^ polymers.

As the degree of polymerization increases, the state of pluronic polymers ranges from liquid to semi-solid to waxy solid. Pluronics can be divided into four categories according to differences in their hydrophilic-lipophilic balance (HLB) and PPO chain length (Figure 2): (I) HLB 20–29 hydrophilic pluronics, such as pluronic F68, pluronic F108, and pluronic F127 [52,53,54]; (II) pluronics with a HLB < 20 and PPO chain < 30, such as pluronic L35, pluronic L44, and pluronic L64 [55,56,57]; (III) pluronics with a HLB < 20 and PPO chain of 30–60, such as pluronic P85, pluronic P105, and pluronic L61 [58,59,60]; (IV) pluronics with a HLB < 20 and a PPO chain > 60, such as pluronic P123 and pluronic L121 [50,61].

The length of PEO and PPO relative to the HLB value significantly affects the particle size, drug loading, stability, and drug release profile of the pluronic polymer [62]. On one hand, the loading capacity of micelles increases as the length of the hydrophobic portion increases, but the colloidal stability of nanoparticles with low HLB values may decrease, falling outside the preferred size range for drug delivery, which was 40–50 nm [14]. On the other hand, when the length of the hydrophilic portion increases, the loading capacity of micelles may decrease, but, above the critical micelle concentration range, the colloidal stability of nanoparticles may improve. Pluronic micelles are always used in drug delivery systems in conjunction with other stimuli-responsive materials to improve their drug loading efficiency and stability.

### 2.1. HLB (Hydrophile-Lipophile Balance) 20–29 Hydrophilic Pluronics

The first class of pluronic polymers includes F68, F108, and F127. These not only have good hydrophilicity, but also have excellent biocompatibility [63]. Therefore, their properties are beneficial for the accumulation of drugs in the organism and the prolongation of the blood circulation time [64]. In addition, F127 is the most widely polymer used due to its diverse nature. Therefore, the thermo-reversible gel properties of F127 show improved retention and have major potential in pharmaceutical applications [65]. Furthermore, F127 can be used as a nano stabilizer to prevent aggregation of nanoparticles, and so on.

**Figure 3:**
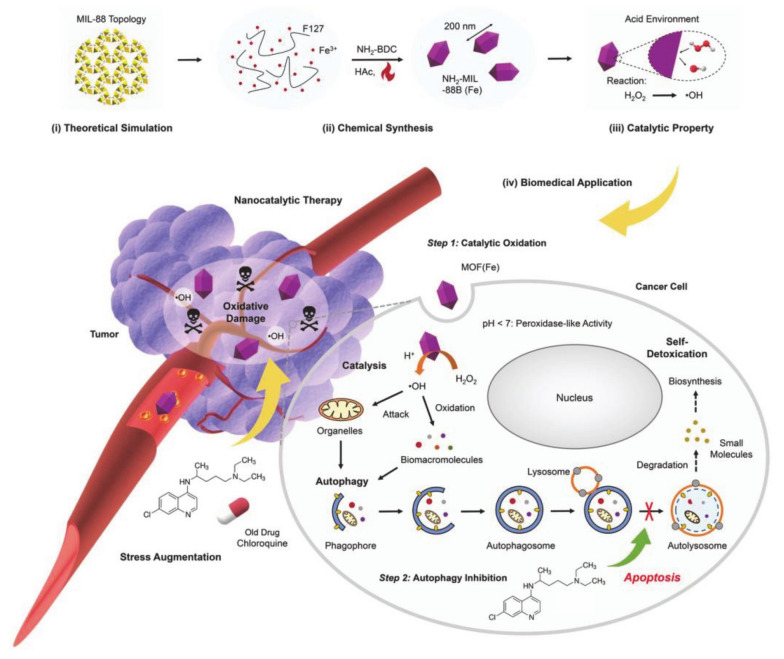
Scheme of the underlying material chemistry and therapeutic concept behind the use of pluronic polymers as a therapy to increase tumor-specific oxidative damage. Step 1: MOF (Fe) catalyzes Fenton-like reactions in cancer cells to result in highly oxidative •OH, which inactivates the protein. Step 2: CQ is deployed to inhibit autophagy by deacidifying lysosome and blocking the fusion of autophagosomes with lysosomes, thus preventing cargo degradation (reproduced with permission from Yang et al. 2020) [66]. Copyright @2020 WILEY-VCH).

Yang et al. [66] proposed a combined treatment method that simultaneously inhibited autophagy and enhanced nanocatalytic cancer therapy by using pharmacological modulators to enhance ROS-induced oxidative damage in synergistic cancer therapy (Figure 3). Thus, a nano-scale metal organic framework (MOF) with inherent peroxidase activity, named NH_2_-MIL-88B (Fe), was synthesized by a hydrothermal method with Fe(III) salt and 2-aminoterephthalic acid (NH_2_-BDC), and this was then used to trigger the entire treatment process. The iron-containing MOF [MOF(Fe)] nanocatalyst (peroxidase mimic) with F127 wrapped triggers a Fenton-like reaction in cancer cells, resulting in a disproportionate amount of H_2_O_2_ and a highly toxic level of •OH, which triggers oxidation that inactivates the protein. At the same time, chloroquine (CQ), as an effective autophagy inhibitor, can be used to deacidify lysosomes and inhibit autophagy. Autophagy protects cancer cells from damage by toxic proteins. All of these elements combine to form a strategy that has a synergistic effect and is suitable for amplifying tumor-specific oxidative damage. Among these pluronics, F127 plays an important role as a nano-stabilizer, guaranteeing their subsequent biological application, which can also be used in maintaining electrical neutrality and preventing the aggregation of nanoparticles in the salt solution.

**Figure 4:**
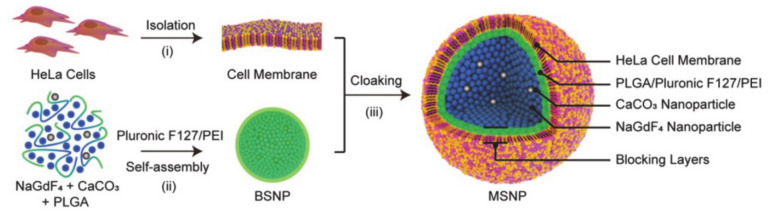
Schematic diagram of cell membrane coated-BSNPs (MSNPs) as a smart contrast agent for T1-weighted MRI, which consists of cell membrane and bare self-assembled nanoparticles (BSNPs) (reproduced with permission from Yang et al. 2020) [66]. Copyright @2020 WILEY-VCH).

Yi et al. [67] designed a magnetic resonance imaging (MRI) nanoplatform with pH responsiveness, high tumor selectivity, and good biocompatibility, which was formed by self-assembled NaGdF_4_-CaCO_3_ encapsulated by pluronic F127 and cell membranes (Figure 4). Nanoparticles based on the Gd^3+^ series are effective imaging agents, but have short circulation time in the blood and lack specificity in vivo, which has reduced their suitability for tracking and monitoring the tumor area. The CaCO_3_ nanoparticles are sensitive to the weakly acidic environment of the tumor, so the cell membrane and pluronic F127 are used to encapsulate the nano-platform. The introduction of F127 can be utilized to delay the rapid renal clearance, hence F127 is beneficial for extending the cycle time of the nanoparticles and enhancing stability. It can be applied in real-time tracking and visualization. In short, the combination of NaGdF_4_ and CaCO_3_ nanoparticles could be disassociated under acidic conditions, while the biomimetic cell membrane surface of the nanosystem enhances biological stability, prolonging circulation and tumor targeting, which results in a more selective MRI contrast agent. In light of these findings, it is clear that pluronic-based drug delivery systems have great advantages as dual-responsive cancer treatments.

**Figure 5:**
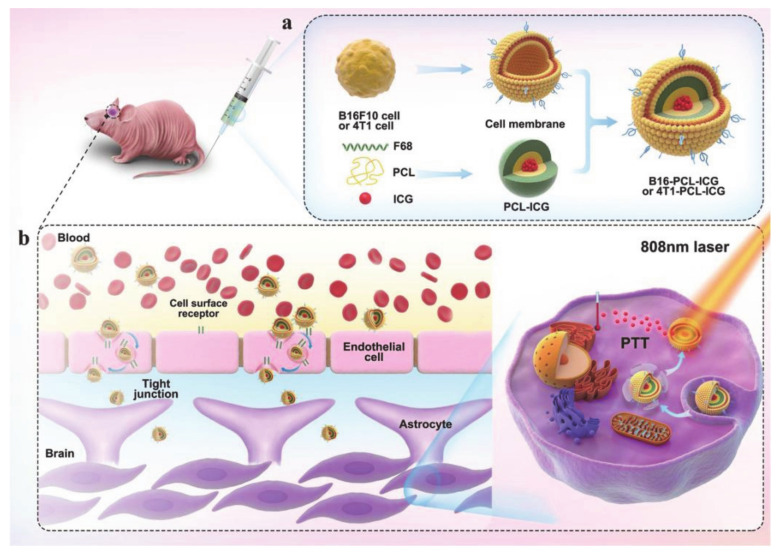
(**a**) Preparation process of biomimetic nanocarriers. (**b**) The possible mechanism of biomimetic nanocarriers across the BBB to brain tumors for imaging and PTT (reproduced with permission from Wang et al. 2020 [68] Copyright @2020 WILEY-VCH).

Wang et al. [68] designed a bionic nanocarrier based on the cell membrane of brain metastases, which could be used for imaging and photothermal therapy (PTT) (Figure 5). The biomimetic nanocarrier consists of two components: the core is made of polymer nanoparticles (NPs) loaded with indocyanine green (ICG) prepared from polycaprolactone (PCL) and pluronic copolymer F68, which functions as an imaging agent and a photothermal agent, while the shell is a brain metastatic tumor cell membrane extracted from a cancer cell. The amphiphilic F68 is used to wrap the ICG and give it hydrophilicity, so that it can be smoothly encapsulated by the cell membrane. The F68 forms nanoparticles via self-assembly in water, which enhances solubility, biocompatibility, and easy transportation. The entry and exit of brain materials is controlled by the blood-brain barrier (BBB), so a large number of drugs are excluded from the brain, making it difficult to treat brain tumors. However, by mimicking the source cells, biomimetic nanocarriers help to enhance the permeability of the BBB, prolong drug circulation time, and facilitate the accumulation of drugs in tumors. Hence, nanoparticles constructed by using brain metastatic tumor cell membrane as a camouflage have a higher ability to cross the BBB and destroy the BBB, thereby allowing for fluorescence imaging and PTT treatment of early brain tumors. In conclusion, pluronic-based drug delivery systems have many bioimaging applications and represent useful candidates for radiation-responsive cancer treatment.

**Figure 6:**
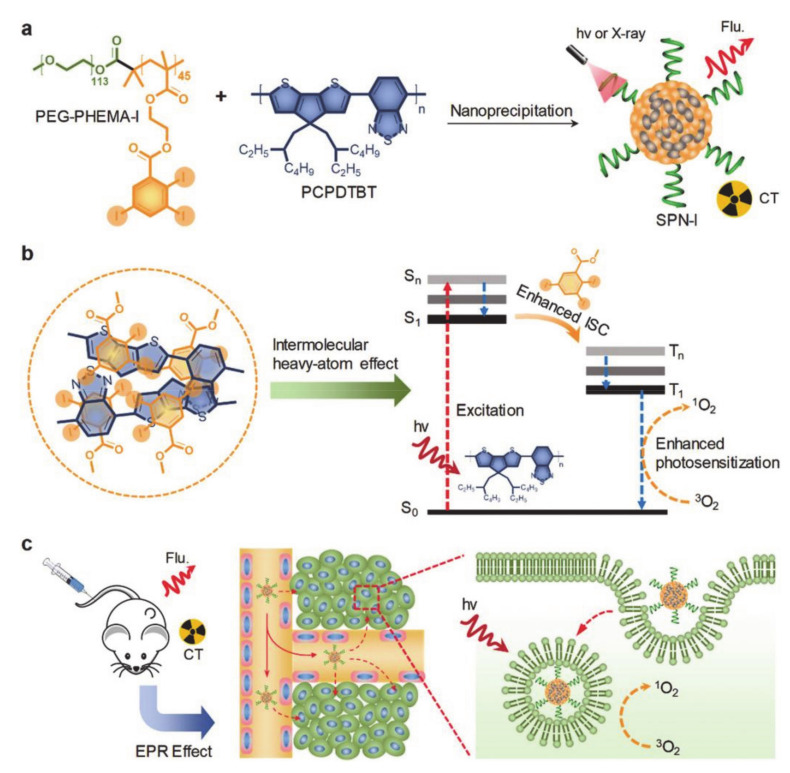
(**a**) The preparation of SPN-I. (**b**) The mechanism of enhanced PS by an iodine-induced heavy-atom effect. (**c**) The CT/fluorescence dual-modal imaging guided PDT of cancer in vivo (reproduced with permission from Zhou et al. 2020 [69]. Copyright @2020 WILEY-VCH).

Zhou et al. [69] designed an iodine-rich SPN-based nanotheranostic (SPN-I) for photodynamic therapy (PDT). The near-infrared (NIR) absorbing semiconducting polymer (PCPDTBT) and F127 encapsulated by the iodine grafted amphiphilic copolymer (PEG-PHEMA-I) were co-precipitated to obtain SPN-1 nanoparticles (Figure 6a). Among them, PCPDTBT was used as the source of the NIR fluorescence signal and photosensitizer. Moreover, the iodine in the PEG-PHEMA-I can be used not only to provide a high X-ray attenuation coefficient, but can also be applied to cause an intramolecular and intermolecular heavy atom effect (HAE), thereby increasing the intersystem crossing (ISC) rate and inducing more ROS generation, which in turn increases the ^1^O_2_ quantum yield (Figure 6b). F127 is suitable for the formation of uniform nanoparticles and improves biocompatibility. Figure 6c shows the mechanism of SPN-I for computed tomography (CT) and fluorescence double-peak imaging. It has the advantages of good stability, deep tissue penetration, and high sensitivity. In short, the introduction of iodine in this review could greatly improve the efficiency of PDT and tumor suppression without increasing side effects.

**Figure 7:**
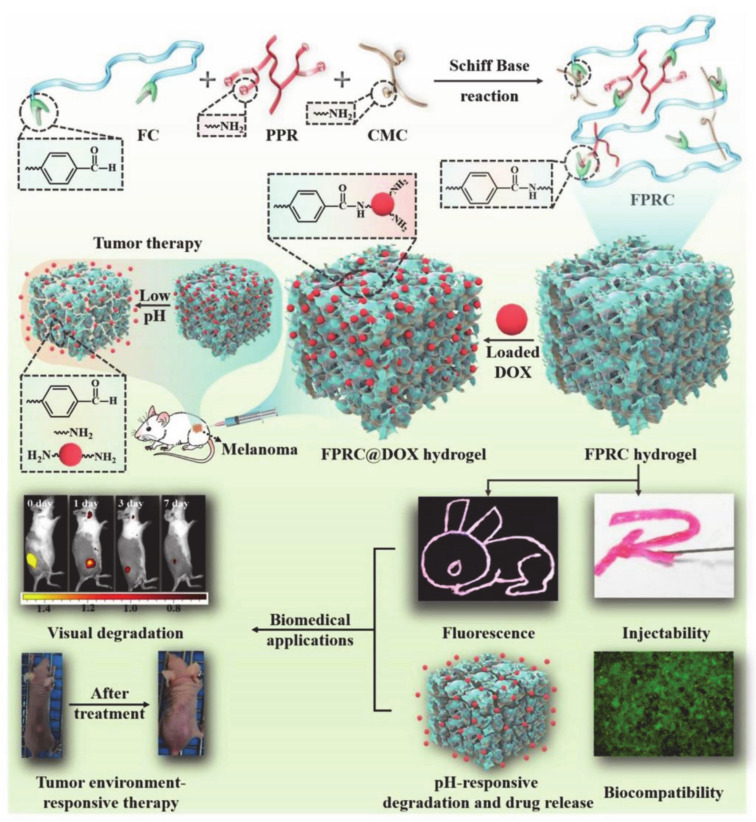
The synthesis process of multifunctional FPRC hydrogel and the effective application of FPRC@DOX hydrogel in treatment (reproduced with permission from Wang et al. 2020 [70]. Copyright @2020 Elsevier Ltd.).

Wang et al. [70] designed and prepared a multifunctional injectable biodegradable-citric acid-based self-healing scaffold, named the F127-CHO(FC)-PPR-CMC hybrid hydrogel system (FPRC hydrogel), which can be used for visual biodegradation tracking and microenvironmental responsive tumor therapy (Figure 7). Red fluorescence-emissive polycitrate-polymine-rhodamine B polymer (PPR) is a type of citric acid polymer that is resistant to deformation, which allows for controlled biodegradation, and has a low cost. Carboxymethyl chitosan (CMC) provides excellent stability and biocompatibility. In the study by Min Wang et al., F127 was used as a drug carrier due to its low toxicity and reverse thermal gel properties. CMC gave the scaffolds the properties of heat sensitivity, injectability, and a self-healing ability. In addition, FPRC hydrogels have high-efficiency DOX insertion and typical pH-responsive DOX release. The FPRC scaffolds not only have pH-responsive degradation/drug release to doxorubicin (DOX), but also have good photostability and in vivo permeability, which could be useful for in vivo tracking. Therefore, when compared with free DOX, the use of DOX in combination with a FPRC scaffold could inhibit tumor growth more effectively. All in all, the FPRC scaffolds exhibit a high therapeutic effect and low biological toxicity.

**Figure 8:**
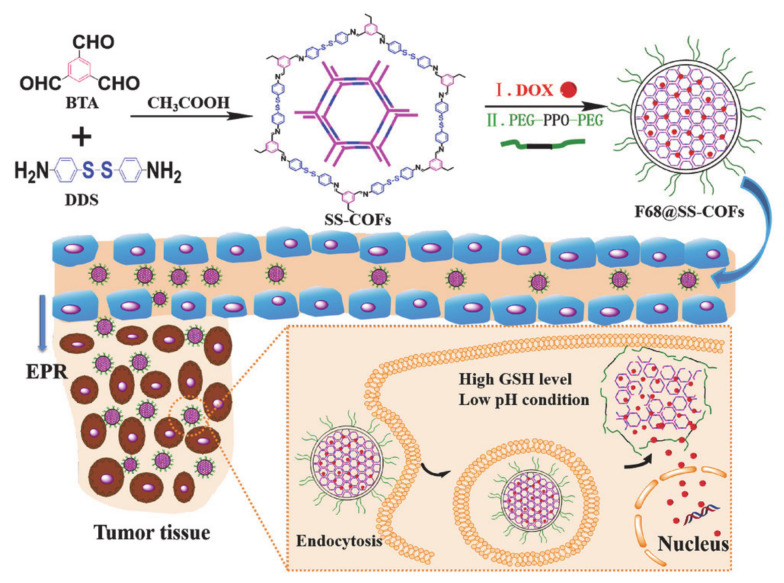
The preparation of drug-loaded F68@SS-COFs and their GSH-responsive drug release (reproduced with permission from Liu et al. 2020 [71]. Copyright @2020 WILEY-VCH).

Liu et al. [71] designed and synthesized a pluronic F68 encapsulated PEGylated redox-responsive nano-scale covalent organic framework (COF denoted as F68@SS-COF) for efficient loading and delivery of DOX (Figure 8). F68@SS-COF was prepared via self-assembly of pluronic F68 and COF with disulfide bonds derived from the universal Schiff base reaction between disulfide bonds. Then, DOX was effectively encapsulated through hydrophobic interaction and π-π stacking interaction, which is attributed to the large pore surface area of COF that can achieve high drug loading and good biocompatibility. At the same time, disulfide bonds make it responsive to GSH over-expression in tumor cells, thereby effectively releasing DOX to kill tumor cells and achieving a high loading rate and very low premature leakage. In addition, F68 can not only be used to enhance the stability of nanoparticles, it can also improve the uncontrolled size of COF, dissociation caused by dilution, and premature drug leakage. Moreover, the PEG chain of F68 has the effect of increasing stability and prolonging blood circulation. This strategy represents an excellent option for tumor treatment with redox responsiveness.

**Figure 9:**
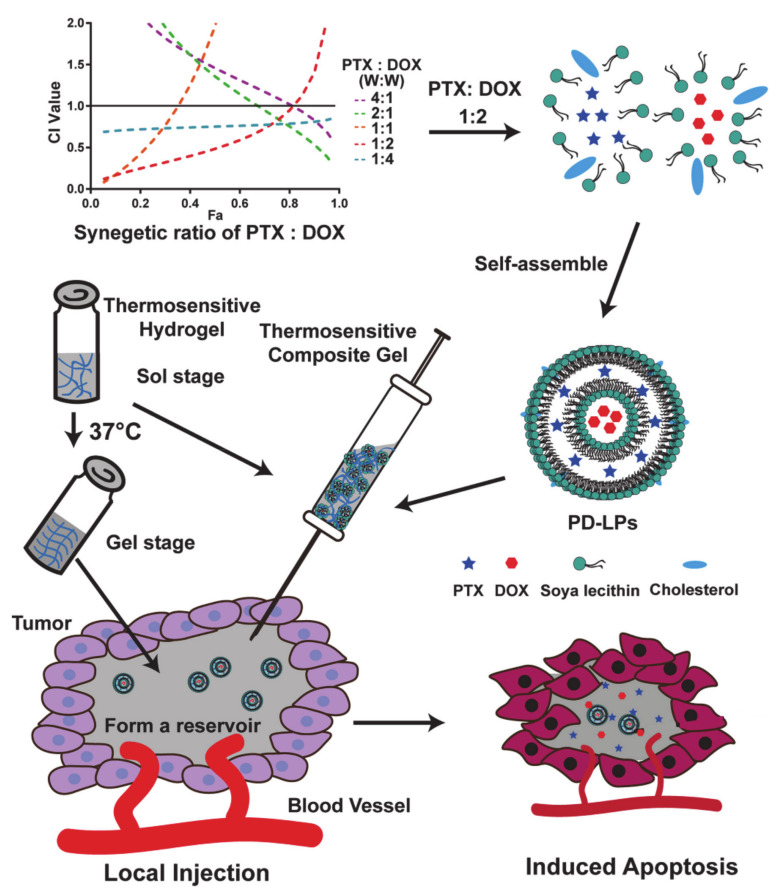
The preparation of PD-LPs Gel with the optimal synergistic ratio (Fa: the changes in the drug effect) (reproduced with permission from Fu et al. 2019 [72]. Copyright @2019 Elsevier Inc.).

Fu et al. [72] designed and prepared a multi-drug loaded liposomes system (PD-LPs) by encapsulating PTX and DOX in liposomes at an optimal ratio, and combining them with a heat-sensitive hydrogel (Gel) which was composing of pluronic F127, sodium alginate (SA), and hydroxypropyl methylcellulose (HPMC) to prepare the PD-LPs Gel (Figure 9). However, the poor solubility of PTX and the strong cytotoxicity of DOX limits the application of this system in tumor areas. In addition, the synergy of PTX and DOX did not have cross-resistance and was seen to induce mitochondrial apoptosis. Therefore, the introduction of a pluronic polymer allowed it to act as not only a drug carrier, but also resulted in an enhanced EPR effect and increased accumulation in tumor areas. Moreover, F127 was applied to heat-sensitive gels in this system, which allowed it to target tumors and maintain drug concentration. The PD-LPs Gel can maintain effective drug concentration at the tumor site by forming a reservoir at the tumor, and has excellent biocompatibility and drug targeting. This thermosensitive nanocomposite gel could increase the concentration of the drug, prolong the retention time of the drug at the tumor area, has low systemic toxicity, and achieves commendable therapeutic effects.

**Figure 10:**
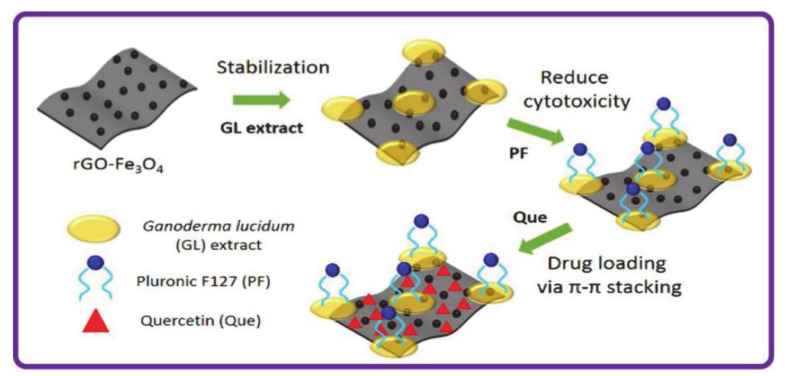
The preparation and drug delivery of superparamagnetic graphene-based nanocomposites (reproduced with permission from Lee et al. 2020 [73]. Copyright @2020 Elsevier B.V.).

Lee et al. [73] designed and synthesized superparamagnetic graphene-based magnetite nanocomposites (rGO-Fe_3_O_4_) for targeted drug delivery, and used Ganoderma lucidum extract (GL) to stabilize rGO-Fe_3_O_4_, improving the water dispersion and stability of the nanocomposite. The introduction of pluronic F127 (PF) not only gave the composite materials a wider range of biological applications, but also reduced the overall cytotoxicity and improved biocompatibility (Figure 10). The nanomaterial was then used to encapsulate the natural anticancer drug Quercetin (Que) through π-π stacking and hydrophobic interaction to obtain rGO-Fe_3_O_4_-GL-PF. The Fe_3_O_4_ has low toxicity and superparamagnetic properties, so it can be used for targeted drug delivery. In addition, the introduction of GL could improve the stability of rGO-Fe_3_O_4_ and the agglomeration of nanoparticles. Overall, rGO-Fe_3_O_4_-GL-PF represents a potential tumor-targeting drug carrier with magnetic responsiveness.

### 2.2. Pluronic with HLB <20 and PO Chain <30

The second class of pluronic polymers includes those such as L64, L44, and L35. Polymers in this class have the smallest PEO and PPO, which not only endows the nanostructures with the ability to self-assemble in aqueous media, but also gives them a more suitable size and a more stable structure [74].

**Figure 11:**
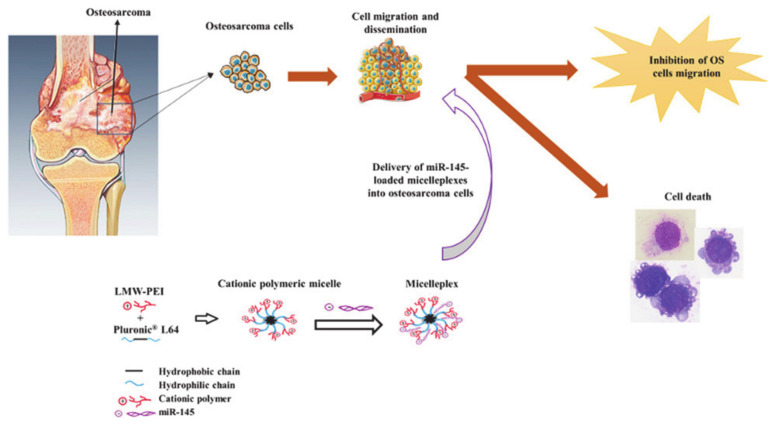
Schematic diagram of the synthesis and mode of action of the micellar complex L64-PEI/miR-145 (reproduced with permission from Magalhães et al. 2018 [75]. Copyright @2018 Elsevier B.V.).

Today, gene therapy is an innovative option for the treatment of osteosarcoma (OS). It involves the transfer of genetic material through a nano system to inhibit or replace the expression of mutant genes. The introduction of miR-145 inhibits cell proliferation and migration, thereby inducing tumor cell apoptosis. Magalhães et al. [75] developed a type of nanosystem (L64-PEI) and used it to deliver miR-145 to obtain micellar complexes (L64-PEI/miR-145) (Figure 11). Among them, pluronic L64 endowed the nanostructure with the ability to self-assemble in an aqueous medium, and had a more suitable size and a more stable structure. In addition, the hydrophilic shell of L64 allowed it to avoid non-specific capture by the reticuloendothelial system and thus resulted in higher bioavailability and better biocompatibility. Moreover, PEI helped to increase cellular uptake through the endocytic pathway. In short, pluronic-based drug delivery systems allow the effective transport of genetic material to promote apoptosis and necrosis of cancer cells, which provides new options for gene therapy.

He et al. [76] developed an intramuscular gene delivery method by combining pluronic L64 and an optimized electric pulse to form an electric pulse system to compress and protect plasmid DNA (pDNA). The interaction of pluronic L64 with the cell membrane enhances its permeability, thereby promoting the migration of pDNA across the permeable membrane. In addition, L64 also helped accelerate the escape of pDNA from lysosomes and achieved better curative effects. Epigallocatechin gallate (EGCG) is a natural compound that not only has a strong affinity for DNA through cooperative hydrogen bonding and hydrophobic interaction, but can also penetrate or be absorbed into the cell membrane to promote transmembrane transport. Therefore, EGCG could effectively be used to compress and protect pDNA, and it significantly increases the level of transgene expression. In short, pluronic-based drug delivery systems represent a useful delivery system for intramuscular gene delivery, and are expected to have potential for further applications.

**Figure 12:**
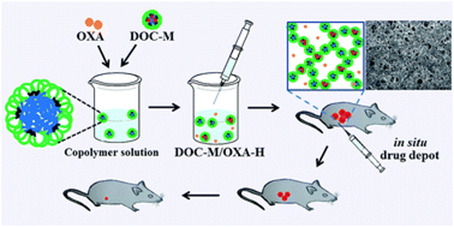
Schematic diagram of the synergistic effect of intratumor injection of dual drug formulations (reproduced with permission from Li et al. 2015 [77]. Copyright @2015 Royal Society of Chemistry).

Li et al. [77] designed a drug delivery system (DOC-M/OXA-H), which was prepared with block copolymer PLLA-L35-PLLA to form a thermosensitive gel (Figure 12). The DOC-M/OXA-H system consists of docetaxel-loaded micelles (DOC-M) and oxaliplatin-loaded hydrogels (OXA-H). DOC is a hydrophobic anticancer drug that can be loaded onto copolymer micelles. At the same time, OXA-H not only confers anti-tumor effects, it also gives micelles the ability to change phase with temperature. DOC-M/OXA-H transforms into a solid gel at physiological temperature through a flowable sol in the environment, and can be used as a drug reservoir to achieve a high curative effect and synergistic drug treatment. The L35 pluronic not only improved the bioavailability and blood circulation time of the gel, but also reduced systemic toxicity, thereby enhancing the effective killing of tumors and resulting in better safety. Clearly, the polymeric drug delivery system based on this pluronic presents a new strategy for the treatment of colon cancer as a controlled dual drug delivery system that forms a gel.

**Figure 13:**
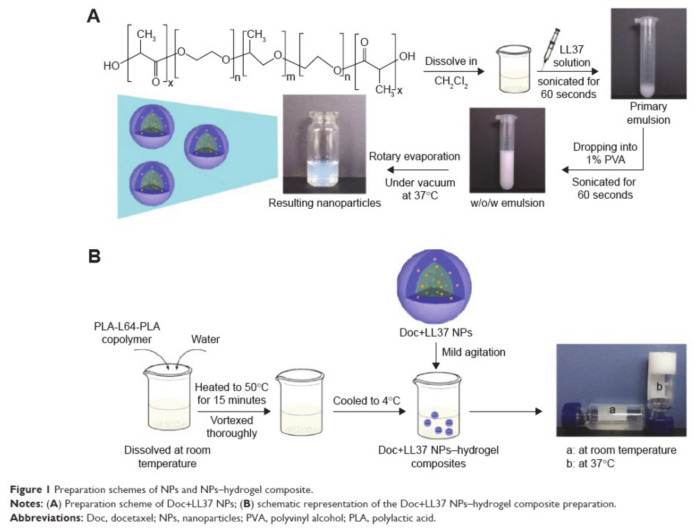
Schematic diagram of the preparation scheme of (**A**) NPs and (**B**) NPs-hydrogel composites (reproduced with permission from Fan et al. 2015 [78]. Copyright @2015 Dove Medical Press).

Synergistic treatment can regulate different signal transduction pathways in cancer cells to enhance efficacy. Fan et al. [78] developed a biodegradable and injectable drug delivery system that co-encapsulated the chemotherapeutic drug docetaxel (Doc) and the LL37 peptide polymer nanoparticles (Doc + LL37 NPs) in a thermosensitive hydrogel system for colorectal cancer therapy (Figure 13). LL37 is an endogenous tumor suppressor peptide that can induce apoptosis of cancer cells, but it does have greater toxic side effects. Therefore, Fan et al. introduced the anti-cancer drug Doc for collaborative treatment in combination with LL37. Intraperitoneal chemotherapy is the best way to treat colorectal peritoneal cancer and the thermosensitive hydrogel PLA-L35-PLA nanoparticles have the advantages of having high biocompatibility and being sensitive to environmental stimuli. Among the nanoparticles, pluronic L35 endows Doc + LL37 NPs with temperature-responsive controlled drug release, with stable quantity and size. The nature of the hydrogel allows for extended treatment time and reduces systemic toxicity. This pluronic-based polymer drug delivery system is expected to achieve better anti-angiogenesis and anti-tumor activity.

### 2.3. Pluronics with HLB < 20 and PO Chain 30–60

The third class of pluronic polymer includes P85, P105, and L61. Their dissolution and self-assembly properties in water are used to load hydrophobic drugs. Moreover, they have the advantage of consuming ATP in MDR cancer cells to inhibit the outflow of P-gp, and also help to prolong the blood circulation time of the drug they are delivering [79,80].

**Figure 14:**
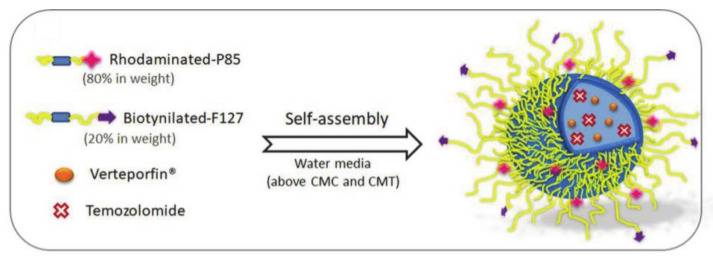
Schematic representation of pluronic P85/F127 m-NP preparation (reproduced with permission from Pellosi et al. 2019 [81]. Copyright @2019 American Chemical Society).

Pellosi et al. [81] developed multifunctional pluronic P85/F127 NPs (hereafter referred to as m-NP) for the treatment of glioblastoma multiforme (GBM). The chemotherapy drug temozolomide (TMZ) and the photosensitizer verteporfin (VP) were loaded onto the NPs to achieve a synergistic effect of both biological therapy and PDT (Figure 14). Pluronics can self-assemble into core-shell NPs in water, loaded with hydrophobic drugs, protecting these drugs from degradation and renal clearance, thus improving the selectivity of these drugs for tumors through enhanced permeability and retention (EPR). Among them, biotinylated F127 has good biocompatibility, so it can be absorbed by cancer cells and even glioblastoma cells. However, as already mentioned, the BBB controls the entry and exit of substances to the brain, making it difficult to treat brain tumors. It is worth mentioning that P85 can effectively hypersensitize P-glycoprotein and surpass the BBB, thereby increasing selective uptake by cancer cells. Pluronic-based polymer drug delivery systems have played an important role in effectively treating GBM through targeted PDT/chemotherapy combination treatment strategies.

Anti-tumor drugs often cause systemic toxicity due to their inability to target tumor cells exclusively, and due to limited access to the brain due to the BBB and the blood-brain tumor barrier (BBTB). In order to enhance the treatment of brain tumors, it is necessary to overcome both the BBB and the BBTB. Niu et al. [82] designed and prepared dual-targeting DOX micelles (GF-DOX) based on the pluronic P105 polymer functionalized with glucose and folic acid for enhanced brain distribution. The glucose transporter 1 (GLUT1) is highly concentrated in the cerebral blood vessels, and so the glucose in these micelles not only meets the energy needs of the brain, but can also effectively pass through the BBB by promoting GLUT1. At the same time, folate receptors (FRs) are overexpressed in cancer cells and therefore are suitable for brain-targeting drugs. In addition, P105 is suited for ATP consumption in MDR cancer cells to inhibit P-gp outflow, promoting the dual-targeting DOX micelles to release drugs in the brain. This pluronic-based polymer drug delivery system can provide new ideas for dual-targeted drugs and better anti-tumor treatments.

**Figure 15:**
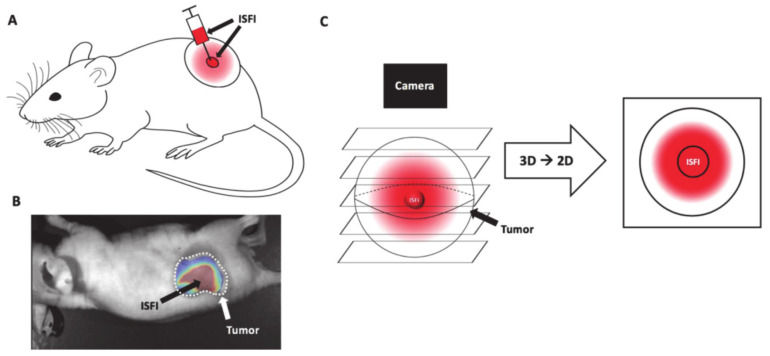
(**A**) Intratumoral ISFI injection into a subcutaneous murine tumor. (**B**) Intratumoral Dox loaded ISFI (red) fuorescent image. (**C**) Schematic of Dox distribution above and below ISFI (reproduced with permission from Jeganathan et al. 2020 [83]. Copyright @2020 The Author(s)).

Jeganathan et al. [83] designed and developed an in situ forming implant (ISFI) that locally delivers P-gp inhibitors and chemotherapeutics through minimally invasive injection. ISFI is a polymer drug solution that undergoes a phase change after being injected into an aqueous environment (such as a tumor), which can target tumor cells with high concentrations of drugs without the usual associated systemic toxicity. Jeganathan et al. converted ISFI into a solid reservoir to release the P-gp inhibitor pluronic P85 or Valspodar (Val), and used the chemotherapy drug Dox to overcome P-gp resistance (Figure 15A). P85 was able to overcome MDR by regulating the expression and function of P-gp. It also helped to prolong circulation time in the body and accumulation in the tumor area. Moreover, Val competitively bound with the drugs in P-gp. The combined administration of Dox and Val could increase the accumulation of drugs and inhibit tumor growth, and ISFI shows great potential as a treatment option for local delivery of drugs without systemic toxicity.

**Figure 16:**
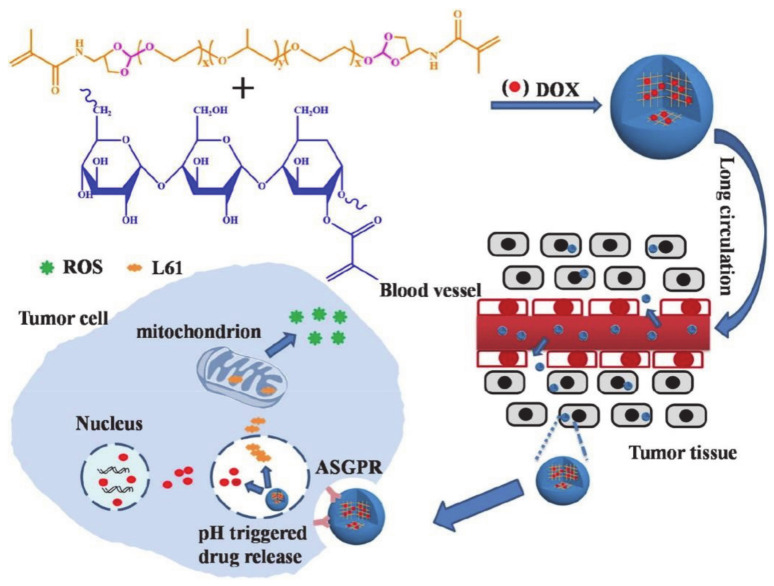
The preparation and drug delivery of DOX-loaded pullulan nanogels (reproduced with permission from Zheng et al. 2019 [84]. Copyright @2019 Elsevier B.V.).

Zheng et al. [84] designed a series of pH-responsive nanogels that are formed by crosslinking natural polymers with orthoester-based crosslinking agents (Figure 16). The authors prepared two kinds of nanogels with a methacrylated pullulan (Pullulan-M), crosslinking agent, acid-insensitive methacrylated copolymer (L61-M), or acid-labile orthoester modified pluronic polymer (L61-MOE). Pul-L61-MOE, which was sensitive to pH, showed a time-dependent degradation and a high drug release rate. In addition, the endocytosis mediated by the lectin asialoglycoprotein receptor (ASGPR) allows for easy entry into tumor cells and actively targets DOX. Pluronic L61 not only endows the nanogel with good biocompatibility, but also has a long blood circulation time and does not damage normal tissues. Moreover, L61 can be used to target mitochondria inducing ROS production, thereby achieving a synergistic anti-tumor effect with DOX targeted therapy. With all this in mind, the polymeric drug delivery system based on pluronic polymers shows excellent potential for use in pH-responsive nanogels and targeted tumor therapy.

### 2.4. Pluronics with HLB < 20 and PO Chain > 60

The fourth class of pluronic polymers includes P123 and L121. Because they have the longest PO chain, their hydrophobicity is greater and they are most widely used as carriers for loading drugs. Moreover, they also have the advantage of consuming adenosine triphosphate (ATP) in multidrug-resistant (MDR) cancer cells, which inhibits the outflow of P glycoprotein (P-gp). Moreover, hydrophobic micelles such as those formed by this type of pluronic polymer may have higher solubilizing power than hydrophilic micelles [85,86].

**Figure 17:**
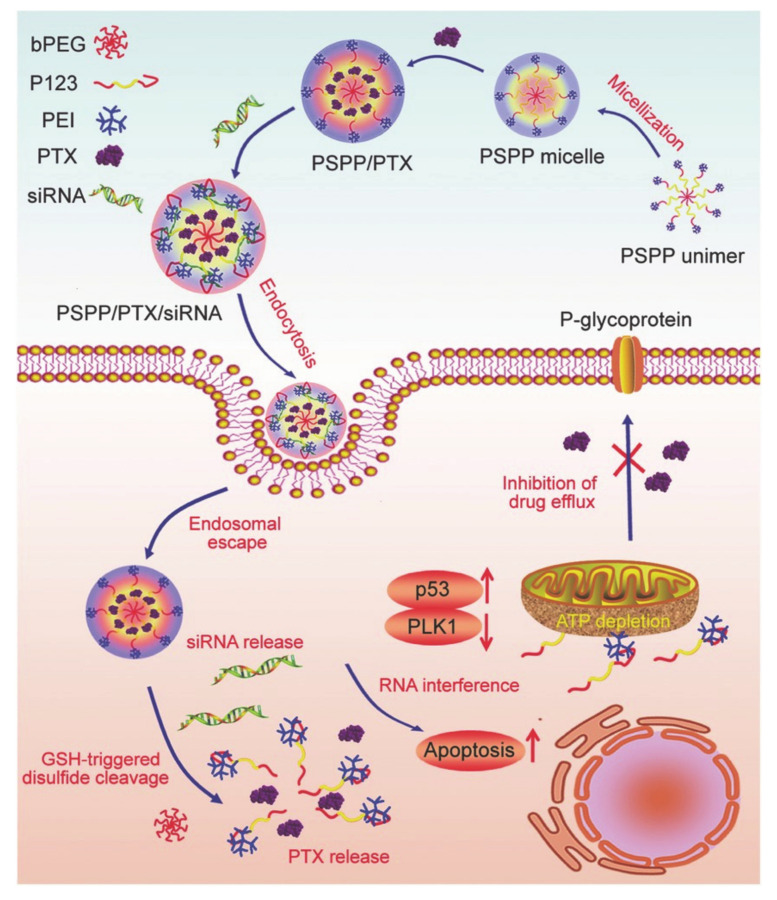
Scheme of the codelivery of siRNA and PTX mediated by PSPP micelles (reproduced with permission from Wang et al. 2017 [87]. Copyright @2017 WILEY-VCH).

Wang et al. [87] designed a redox reaction type, pluronic-based cationic multi-block copolymer micelles (called bPEG-SS-P123-PEI (PSPP)). Under CMC, it self-assembles into cationic micelles, then encapsulated paclitaxel (PTX) and siRNA through hydrophobic interactions and electrostatic interactions to form cationic PSPP/PTX/siRNA micelles, which contain glutathione (GSH) responsiveness (Figure 17). The PEI block can be used to modify P123 (referred to as PEI-P123) to provide cationic properties, and bPEG was used to form a sterically stabilized protective network of micelles capable of crossing the physiological barrier. In addition, P123 was not only used as a drug carrier, it was also used for rapid adenosine triphosphate (ATP) depletion where it effectively reverses MDR both in vivo and in vitro. Therefore, the cleavage of disulfide bonds in the GSH-rich reducing environment of the tumor cells not only promotes the release of PTX and siRNA, but also activates the depletion of ATP. The rapid ATP depletion and RNAi binding induce apoptosis, providing a more effective dual regulation of MDR, which can more effectively inhibit tumor growth. This pluronic-based drug delivery system represents a useful treatment option for effective redox sensitive cancers and to overcome MDR.

**Figure 18:**
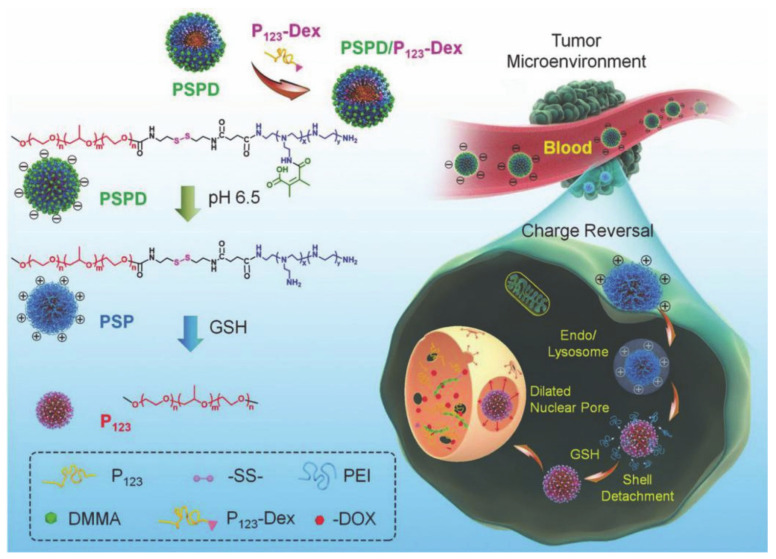
Scheme of the cooperative strategy for drug delivery mediated by hybrid micelle PSPD/P123-Dex (reproduced with permission from Wang et al. 2017 [88]. Copyright @2017 WILEY-VCH).

Wang et al. [88] also designed polymerized mixed micelles that are composed of an amphiphilic polymers that are redox-responsive and a pH-responsive amphiphilic polymer containing Dex. The Dex induces nuclear pore expansion and nuclear targeting during its translocation process. The amphiphilic block (termed P123), which was coupled to a charge reversible block (termed DMMA-PEI) via a disulfide bond (termed as PSPD), is used for pH and redox-triggered structural variation, and can be used to adjust the size of hybrid micelles. The Dex-conjugated P123 amphiphilic block (called P123-Dex) is therefore used to target the nucleus and expand the nuclear pore. The hybrid micelles produced by the two parts are called PSPD/Dex-P123, and are loaded with DOX via a hydrophobic interaction before delivery to the nucleus. P123-Dex’s ability to expand nuclear pores and the dual response of PSPD to achieve a gradual reduction in size represents a new strategy for nuclear targeted drug delivery systems (Figure 18).

**Figure 19:**
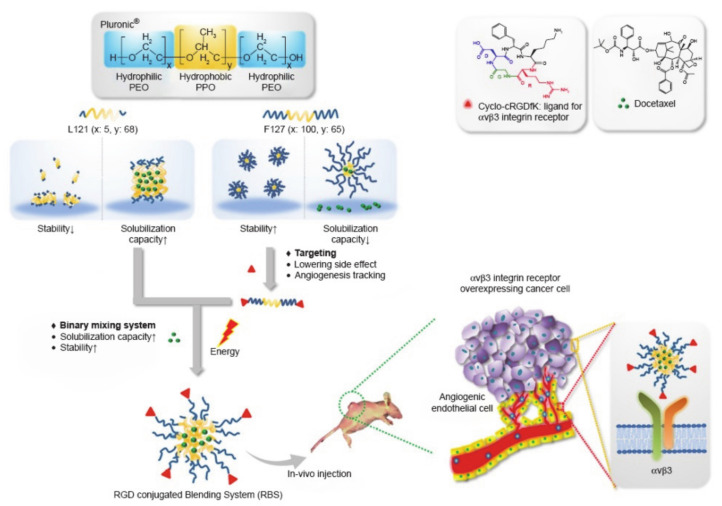
Schematic diagram of cRGD conjugated L121/F127 mixed micelle system (reproduced with permission from Lim et al. 2018 [89]. Copyright @2018 Dove Medical Press).

Lim et al. [89] designed and developed a hybrid system based on pluronic polymer L121/F127 loaded with the anticancer drug docetaxel (DTX) to targeted tumors for treatment (Figure 19). Cyclic arginine-glycine-aspartate tripeptide (cRGD) is an active tumor targeting ligand that was conjugated to nanoparticles and provides several benefits, including its ability to increase cellular uptake in cancer cells, increase anti-cancer activity, and aid in effective drug accumulation, resulting from the binding of cRGD to proteins that are overexpressed in tumor tissues. Among them, the L121/F127 blending system helped to obtain a suitable size and better colloidal stability. Pluronic L121/F127 could be used to load more hydrophobic drugs by forming a more suitable size. In addition, it could also be applied to help improve colloidal stability. In short, pluronic-based polymer drug delivery systems are expected to encapsulate various drugs by changing the proportion of polymer blends, and have great potential for improving anti-tumor activity.

**Figure 20:**
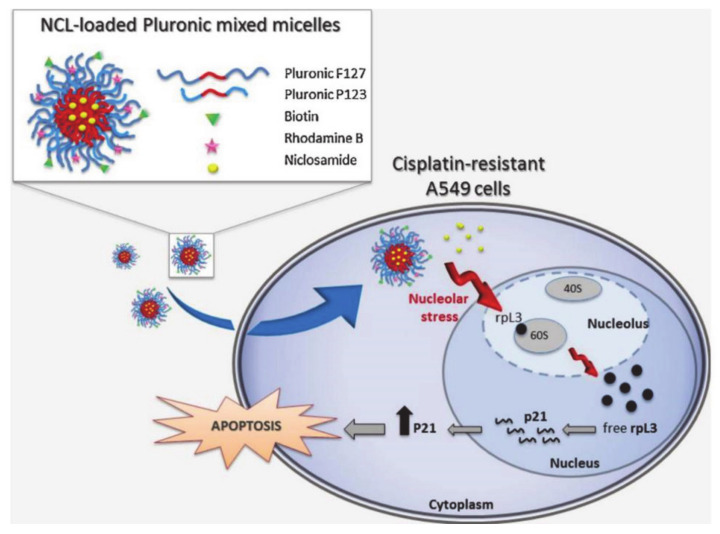
Schematic diagram of the synthesis and mode of action of NCL-loaded pluronic mixed micelles (reproduced with permission from Russo et al. 2016 [90]. Copyright @2016 Elsevier B.V.).

Russo et al. [90] developed a pluronic multifunctional platform P123/F127 mixed micelle for delivering niclosamide (NCL) to targeted cancer cells (Figure 20). NCL overcame MDR by inhibiting the efflux of ATP, thereby effectively inhibiting cell growth. F127 was conjugated with biotin for internalization, which was targeted to cancer cells with negligible systemic toxicity. Rhodamine B modified in P123 was used to label micelles to achieve cell imaging. Moreover, the self-assembly of P123/F127 into micelles can be used to enhance the solubility and biocompatibility of NCL. In addition, P123/F127 can also provide small nanostructures that are applied to prolong drug circulation time and overcome the drug outflow of MDR, thus increasing drug accumulation. These pluronic-based drug delivery systems have great potential for use in the treatment of multi-drug resistant cancers and are expected to achieve the goal of targeted and sustained drug release.

**Figure 21:**
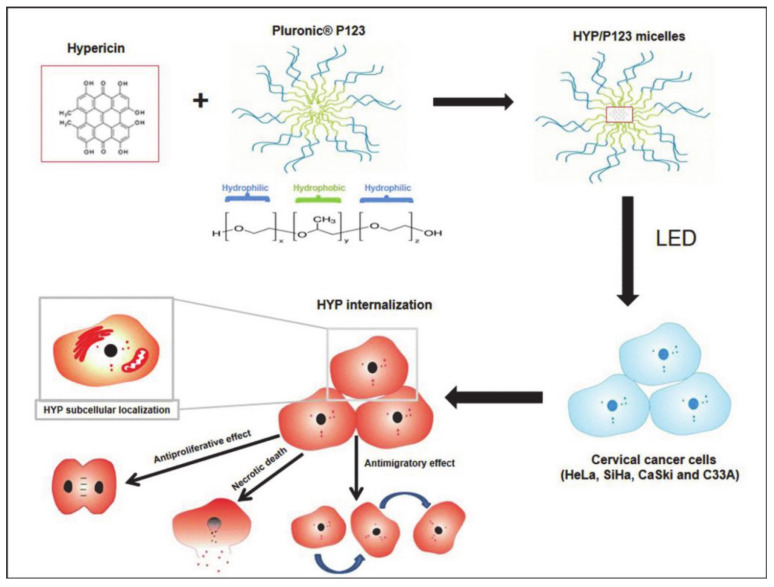
Schematic diagram of the preparation of HYP/P123 and its treatment (reproduced with permission from Damke et al. 2020 [91]. Copyright @2020 Elsevier Inc).

Damke et al. [91] developed micelles (HYP/P123) of pluronic P123 encapsulating hypericin (HYP) for PDT (Figure 21). As a natural photosensitizer (PS), HYP not only has anti-proliferation and cytotoxic effects, it also has high selectivity for tumors. However, the high hydrophobicity of HYP reduces the production of ^1^O_2_ and hinders the efficacy of PDT. P123 was introduced herein for the preparation of micelles in order to improve the poor solubility of HYP. The resulting mixture easily self-assembled to form micelles in an aqueous medium, and the inner core was used to load photosensitizer. In addition, P123 is non-toxic, and has good biocompatibility and excellent pharmacokinetics. HYP/P123 is selectively cytotoxic to cervical cancer cells with negligible systemic toxicity. Pluronic-based DDS is expected to become a platform for the treatment of all types of cervical cancer cells.

**Figure 22:**
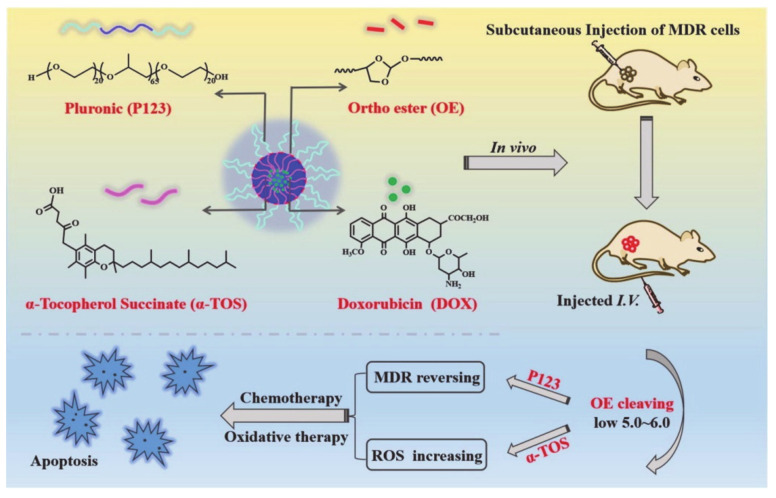
The synthesis and effect of functionalized POT (reproduced with permission from Cheng et al. 2020 [92]. Copyright @2020 Elsevier Inc.).

Cheng, et al. [92] designed a new type of pH-sensitive pluronic micelle that can not only be used for oxidative therapy, but can also reverse the synergistic effects of MDR, which hinders cancer treatment (Figure 22). Through the acid-labile orthoester (OE) bond, the orthoester in particular has better biocompatibility and stability. The authors also used α-tocopherol succinate (α-TOS) to synthesize modified pluronic (P123), which had the function of mediating drug outflow to significantly reverse MDR. At the same time, α-TOS can be used for solubilization to obtain a pH-sensitive copolymer (POT). POT micelles have acid-triggered sensitivity and can be used to target drug release in the tumor area and can also be used to reverse MDR and induce the generation of more reactive oxygen species (ROS). As a result, POT micelles have a good tumor inhibitory effect. This resulted in better stability and anti-hemodilution effect, and in the accumulation and retention of the drug in MDR cells, thereby increasing cytotoxicity and apoptosis when compared with traditional methods. These findings indicate that pluronic-based drug delivery systems can be effectively used for pH-sensitive cancer treatments that overcome MDR.

## 3. Conclusions

In the past few decades, interest in the application of using pluronic micelles for drug delivery has been based on their biocompatibility, high solubility, and passive accumulation in the tumor area. At the same time, their amphiphilic properties and potential to self-assemble into micelles in aqueous solutions make them a promising drug carrier. Amphiphilic properties endow pluronic polymers with surfactant properties, which make them suitable for improving the utilization of drugs and rendering cancer cells more susceptible to drug damage. The micelles contain hydrophobic PPO fragments that can be used to carry different therapeutic and diagnostic agents. Pluronic polymers or pluronic-based composites are responsive to their environment, which enables them to effectively deliver drugs to their target and control drug release.

Drug delivery systems based on pluronic polymers have the advantages of long blood circulation time, strong cellular uptake, and low cytotoxicity. Since pluronic has a series of advantages, which has different applications in many fields. Hydrophilic pluronics shows excellent biocompatibility that enables them to be utilized as stabilizers and retention agents. Hydrophobic pluronics play an important role in loading drugs. In addition, they have the advantage of consuming ATP in MDR cancer cells to inhibit the outflow of P-gp. The remaining pluronic forms micelles of appropriate size through self-assembly. However, the central aim of drug nanocarriers is to effectively target the tumor area. This is because certain drugs are rapidly metabolized or excreted from the body without achieving drug efficacy, and the stimulus response is only suitable for drug release at a specific location. Stimulus responsiveness includes specific exogenous stimuli (for example, changes in temperature, magnetic field, radiation) or endogenous stimuli (changes in pH or redox). Endogenous stimulus sensitivity plays a role through the tumor microenvironment or pathological conditions. Moreover, exogenous stimulus sensitivity is the targeted delivery of pharmacologically active molecules to the tumor area. A wide range of stimuli can trigger drug release at the right place and time, and diversity response materials can also help to trigger targeted and timely drug release and have great potential in stimulus response systems. The prospects of stimulus responsive nanoparticles based on pluronic polymers are broad and have many advantages in drug delivery. However, they still face many challenges due to various extracellular and intracellular barriers hindering delivery, and more systematic and in-depth research is needed to achieve optimal clinical application.
